# Membrane Interactions of the Peroxisomal Proteins PEX5 and PEX14

**DOI:** 10.3389/fcell.2021.651449

**Published:** 2021-04-16

**Authors:** Stefan Gaussmann, Mohanraj Gopalswamy, Maike Eberhardt, Maren Reuter, Peijian Zou, Wolfgang Schliebs, Ralf Erdmann, Michael Sattler

**Affiliations:** ^1^Bavarian NMR Center, Department Chemie, Technische Universität München, Munich, Germany; ^2^Institute of Structural Biology, Helmholtz Zentrum München, Neuherberg, Germany; ^3^Institute of Biochemistry and Pathobiochemistry, Department of Systems Biology, Faculty of Medicine, Ruhr University Bochum, Bochum, Germany

**Keywords:** peroxisome biogenesis, protein targeting, structural biology, NMR, membrane binding

## Abstract

Human PEX5 and PEX14 are essential components of the peroxisomal translocon, which mediates import of cargo enzymes into peroxisomes. PEX5 is a soluble receptor for cargo enzymes comprised of an N-terminal intrinsically disordered domain (NTD) and a C-terminal tetratricopeptide (TPR) domain, which recognizes peroxisomal targeting signal 1 (PTS1) peptide motif in cargo proteins. The PEX5 NTD harbors multiple WF peptide motifs (WxxxF/Y or related motifs) that are recognized by a small globular domain in the NTD of the membrane-associated protein PEX14. How the PEX5 or PEX14 NTDs bind to the peroxisomal membrane and how the interaction between the two proteins is modulated at the membrane is unknown. Here, we characterize the membrane interactions of the PEX5 NTD and PEX14 NTD *in vitro* by membrane mimicking bicelles and nanodiscs using NMR spectroscopy and isothermal titration calorimetry. The PEX14 NTD weakly interacts with membrane mimicking bicelles with a surface that partially overlaps with the WxxxF/Y binding site. The PEX5 NTD harbors multiple interaction sites with the membrane that involve a number of amphipathic α-helical regions, which include some of the WxxxF/Y-motifs. The partially formed α-helical conformation of these regions is stabilized in the presence of bicelles. Notably, ITC data show that the interaction between the PEX5 and PEX14 NTDs is largely unaffected by the presence of the membrane. The PEX5/PEX14 interaction exhibits similar free binding enthalpies, where reduced binding enthalpy in the presence of bicelles is compensated by a reduced entropy loss. This demonstrates that docking of PEX5 to PEX14 at the membrane does not reduce the overall binding affinity between the two proteins, providing insights into the initial phase of PEX5-PEX14 docking in the assembly of the peroxisome translocon.

## Introduction

Peroxisomes are ubiquitous membrane enveloped organelles of eukaryotic cells involved in various metabolic pathways, including β-fatty acid oxidation and removal of toxic oxidation products (Lazarow and Fujiki, 1985; Erdmann et al., 1997; Wanders, 2004; Wanders and Waterham, 2006). Peroxisome biogenesis depends on a number proteins, the so-called peroxins (Distel et al., 1996; Ma et al., 2011). As peroxisomes lack a protein synthesis machinery, peroxisomal matrix proteins need to be imported into the organelle post-translationally. The majority of these cargo proteins are imported via a peroxisomal targeting signal 1 (PTS1), a conserved C-terminal peptide motif, with SKL as canonical sequence (Gould et al., 1987; Ghosh and Berg, 2010). The soluble peroxisomal receptor PEX5 recognizes the PTS1 motif by a C-terminal tetratricopeptide (TPR) domain (Gatto et al., 2000; Stanley et al., 2006). Cytosolic PEX5 shuttles the cargo protein to the peroxisomal membrane (Dodt and Gould, 1996; Dammai and Subramani, 2001; Erdmann and Schliebs, 2005; Rucktaschel et al., 2011). For this, its intrinsically disordered N-terminal domain (NTD) interacts with the membrane-anchored peroxins PEX14 and PEX13 (Schliebs et al., 1999; Saidowsky et al., 2001; Neufeld et al., 2009; Neuhaus et al., 2014). Subsequently, a transient pore is formed and the cargo is tunneled through the membrane (Erdmann and Schliebs, 2005). This step of membrane passaging has been characterized in *Saccharomyces cerevisiae*, where Pex5p and Pex14p are key components of the protein conducting channel (Meinecke et al., 2010).

In contrast to PEX14, which is an integral membrane protein with a single transmembrane span, PEX5 does not contain a classical transmembrane domain (Emmanouilidis et al., 2016). However, it harbors WxxxF/Y (W1-7) and one LVAEF (W0) motif in the NTD, which bind to the PEX14 NTD and have been hypothesized to potentially mediate membrane interactions (Saidowsky et al., 2001; Emmanouilidis et al., 2016). PEX5 cycles between a soluble and a membrane associated state. While this suggests that PEX5 may be able to interact with the membrane, it still requires a co-factor to maintain it at the membrane (Azevedo and Schliebs, 2006). It is expected that the membrane protein PEX14 localizes PEX5 to the membrane, since the PEX14 NTD is able to bind to all eight WF-like -motifs of PEX5 (Neufeld et al., 2009; Neuhaus et al., 2014; Emmanouilidis et al., 2016).

Although the molecular interactions between PEX14 and PEX5 are known, the mechanism by which the cargo is translocated is still poorly understood (Emmanouilidis et al., 2016). It has been proposed that the PEX14 NTD may recruit PEX5 by binding to the W0 and additional WF motifs and thereby initiates pore formation. Recent studies have proposed that the NTD of PEX14 is located inside the peroxisomal lumen (Neuhaus et al., 2014; Barros-Barbosa et al., 2019), and thus would not be easily available for initiating contacts with the PEX5 NTD in the cytosol. Potentially, PEX5 might be recognized by other parts of the docking complex (such as PEX13) or may be targeted to the peroxisomal membrane by direct binding. It is also conceivable that the PEX14 NTD may be transiently exposed to the cytosol and subsequent to PEX5 binding translocate into the peroxisomal lumen. In any case, a common prerequisite for all these models is a membrane localization of PEX5 and PEX14.

Studying proteins in membrane-like environment can be challenging: For *in vitro* binding studies, the membrane mimic should represent the lipid composition of the native environment and compatible with the experimental approach. The peroxisomal membrane lipid composition of eukaryotic cells is not well characterized. Nevertheless, analysis of peroxisomes from rat liver showed a distribution of 27.5% phosphatidylethanolamine (PE), 56.6% phosphatidylcholine (PC), 4.7% phosphatidylinositol (PI), 3.7% sphingomyelin (SPM), and 3% phosphatidylserine (PS) (Hardeman et al., 1990). The high percentage of almost 60% of phosphatidylcholine is well feasible for studies using solution state NMR spectroscopy, since bicelles composed of 1,2-Dimyristoyl-sn-Glycero-3-Phosphocholine (DMPC) and 1,2-Diheptanoyl-sn-Glycero-3-Phosphocholine (D7PC) are well established and favorable due to the relatively low molecular weight. The bicelles assemble into discoid bilayers, where DMPC forms the planar surface of the disk while the short-chain lipids from D7PC form the curvature on the edges, which leads to the dependency of the disk size on the molar ratio of DMPC to D7PC (q). Isotropic bicelles are typically made by a molecular ratio q ranging between 0.2 and 0.5, where the size is shrinking with the value of q (Marcotte and Auger, 2005; Warschawski et al., 2011; Sommer et al., 2012). NMR spectroscopy benefits from the small molecular size of bicelles in terms of resolution derived from relatively sharp line shapes. On the other hand, the high curvature does not represent the native membrane environment very well. For proteins with transmembrane spans, a better membrane mimic can be achieved using nanodiscs, which consists of a planar lipid bilayer encircled with a membrane scaffold protein (MSP). Recent developments in this field allow the production of smaller nanodiscs with a diameter of 6–8 nm with favorable features for NMR studies (Hagn et al., 2018).

The present study combines NMR spectroscopy and isothermal titration calorimetry to characterize the membrane interaction of the NTDs of PEX5 and PEX14 as central components of the peroxisomal pore and investigates the interaction between the two proteins in solution and in the presence of a membrane mimicking environment, thus providing novel insight into early steps of peroxisomal protein translocation.

## Results

### Conformational Analysis of the PEX5 NTD by NMR Spectroscopy

**FIGURE 1:**
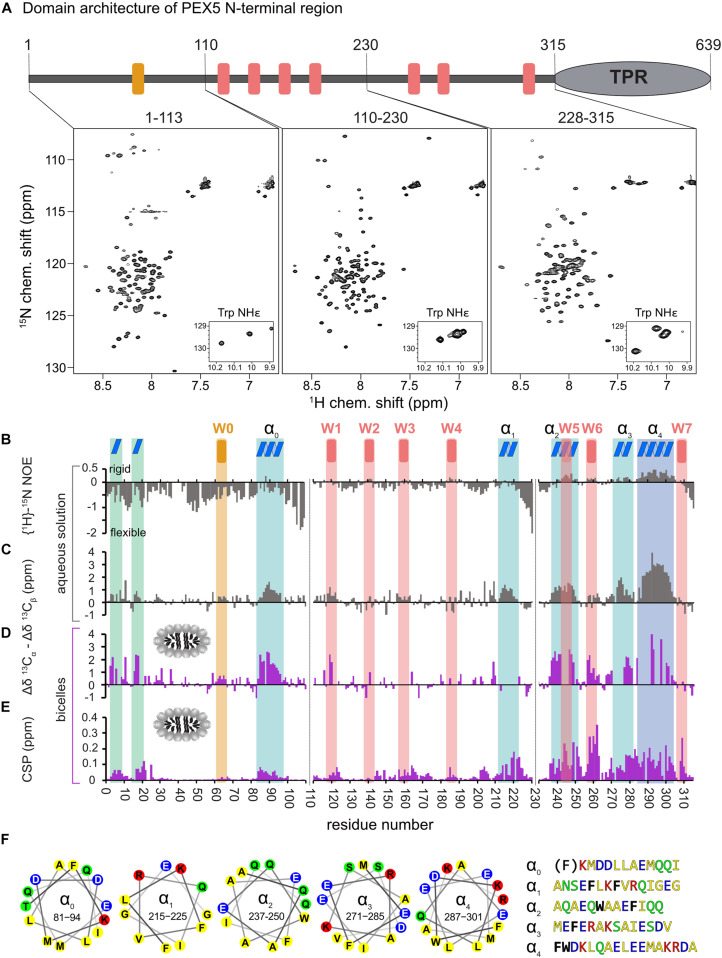
Analysis of PEX5 NTD in the absence and presence of bicelles by NMR spectroscopy. **(A)** PEX5 NTD domain architecture and ^1^H–^15^N HSQC spectra of PEX5 constructs 1–113, 110–230, and 228–315 **(B)**. {^1^H}-^15^N heteronuclear NOE experiments of the three constructs in aqueous solution. Negative values represent a highly flexible peptide backbone conformation. WF and the WF-like motif W0 are indicated by red or orange bars, respectively. **(C)** Less flexible regions were classified as helices α_0_–α_4_ (blue boxes) based on the secondary chemical shift (Δδ^13^C_α_ –Δδ^13^C_β_) **(D)**. In the presence of bicelles the helices and two α-turns located in the first 20 aa, are stabilized which is shown by the secondary chemical shift **(E)**. Chemical shift perturbations extracted from ^1^H-^15^N HSQC experiments demonstrate membrane binding which is mostly mediated by WF6 and the amphipathic helices α_0_–α_4_ visualized as helical wheels in **(F)**. The residues are color coded in yellow for hydrophobic, green for polar, blue for negative charged and red for positive charged sidechains. Phe and Trp residues are shown in black and bold letters with exception of the Phe in α_0_ which does not contribute to the hydrophobic face of the helix.

The N-terminal domain of PEX5 is intrinsically disordered (Emmanouilidis et al., 2016). The NMR spectrum of ^15^N-labeled PEX5 NTD, comprising residues 1–315 (Supplementary Figure 1A) show poor spectral dispersion, with most signals showing amide proton chemical shifts between 8 and 8.5 ppm, typical for intrinsically disordered proteins (IDPs). To reduce signal overlap, we divided the PEX5 NTD into roughly 100 aa long regions with three to four amino acid residues overlapping to the next or previous subconstruct and without disrupting any of the known WF-like motifs. The 2D ^1^H, ^15^N correlation spectra of PEX5 comprising residues 1–113, 110–230, and 228–315 (Figure 1A) show good dispersion and very little signal overlap. Moreover, comparison of the sum of the NMR correlation spectra with the those obtained for the full PEX5 NTD shows little chemical shift differences (Supplementary Figure 1B), suggesting that analysis of the three subregions faithfully reports on the properties in the context of the PEX5 NTD. Next, the residue-specific backbone chemical shifts of the three regions were assigned using standard triple resonance experiments (Sattler et al., 1999), enabling a comprehensive NMR analysis of the PEX5 NTD. This allowed us to analyze the secondary structure and conformational flexibility of the polypeptide backbone, and to map molecular interactions with membrane mimics.

First, we investigated the polypeptide backbone flexibility of the PEX5 NTD using {^1^H}-^15^N heteronuclear NOE experiments for the three NTD subregions. The conformational flexibility of the backbone is reflected by the heteronuclear NOE, with values of ∼0.9 corresponding to a rigid backbone conformation as expected for a globular folded protein (Daragan and Mayo, 1997). The first 110 amino acids of PEX5, including the W0 (LVAEF) motif, shows a highlight flexible backbone conformation, while the remaining 205 amino acids exhibit significantly reduced conformational flexibility. Most of these regions coincide with the WF motifs W1 to W6, with W5 and W6 showing the highest values. This indicates that the region comprising the W5 and W6 motifs is less flexible in solution (Figure 1B).

Next, we analyzed the secondary structure of the PEX5 NTD based on ^13^C_α_ and ^13^C_β_ secondary chemical shifts, which are the difference of chemical shifts compared to those in a random coil conformation (Spera and Bax, 1991). Values around zero indicate random coil while positive and negative values correlate with α-helical and β-strand/extended conformations. Interestingly, the PEX5 NTD shows some regions with α-helical characteristics, which are not related to the WF motifs (Figure 1C). The largest positive secondary chemical shifts are found for residues 285 to 305. Notably for this region also positive heteronuclear NOE values are observed, indicating the presence of a largely formed α helix (α_4_). Four additional regions, residues 81–96 (α_0_), 210–220 (α_1_), 237–250 (α_2_), and 271–285 (α_3_) exhibit transient (partially formed) helical regions indicated by secondary chemical shifts between 1 and 2 ppm (Figure 1C). All these helical regions are very well conserved among eukaryotes and have amphipathic character (Figure 1F), as is well known for the WF motifs. Thus, these regions could mediate protein-protein and/or membrane interaction (Schliebs et al., 1999). Experimental structures have previously shown that W0 and W1 adopt an α-helical fold in the complex with the PEX14 NTD and for some of the PEX5 WF motifs it has been shown that they adopt a partially preformed helix in solution (Neufeld et al., 2009; Neuhaus et al., 2014). Our data show that the WF motifs W5 and W6 are indeed the most helical regions. We identify four additional more transient helices (α_0_–α_4_) that maybe involved in additional interactions.

### PEX5 NTD Interacts With Membranes

NMR membrane binding studies were performed with bicelles consisting of DMPC, a saturated C14:0 lipid. While this is a favorable and well-established membrane mimic for NMR studies it does not reflect the composition of peroxisomal membranes. To assess the validity of using DMPC mimics we performed and compared flotation analyses of PEX5 and PEX5 NTD with liposomes consisting of DMPC and of three volumes 1,2-dioleoyl-*sn*-glycero-3-phosphoethanolamine (DOPE) and seven volumes 1,2-dioleoyl-*sn*-glycero-3-phosphocholine (DOPC). This resembles the composition of 28% phosphoethanolamine and 57% phosphocholine, respectively, of the membranes of rat liver peroxisomes (Hardeman et al., 1990). Consistent with previous observations (Kerssen et al., 2006) a similar fraction of PEX5 was found in association with floated vesicles consisting of DOPE/DOPC (Supplementary Figure 2A). Remarkably, the same flotation behavior was observed with liposomes constituted with DMPC only (Supplementary Figure 2A). Also the PEX5 NTD co-migrated with both floated DMPC and DOPE/DOPC liposomes with comparable efficacy (Supplementary Figure 2B). Taken together, the flotation analyses confirm that the lipids used for mapping the binding sites by NMR are useful and relevant proxies for the lipid binding properties of PEX proteins.

To study potential secondary structure and map the lipid binding regions of the PEX5 NTD we performed NMR titrations with increasing bicelle concentration (Figures 1D,E). We recorded ^15^N correlation spectra in the presence of 0.9 mM bicelles (DMPC/D7PC, q: 0.2). At these concentrations, we observe large chemical shift perturbation and substantial line broadening in the NMR spectra of the PEX5 NTD regions. To confirm and track chemical shift assignments additional triple resonance experiments were recorded in the bicelle-bound state. Comparison of NMR spectra in buffer and in the presence of bicelles reveal significant chemical shift perturbations (CSP) and changes in secondary chemical shifts exclusively for the α-helical regions. We find two small but strongly enhanced helical regions between residues 1 to 20, which show very low helical propensity in aqueous solution and were therefore not classified as preformed helical motifs (Figure 1D). These induced helical motifs are well conserved in sequence and contribute to PEX5 membrane interaction as can be judged from the chemical shift perturbations (Figure 1E). A notable increase in helical propensity is also observed for the helical regions α_0_, α_2_, α_3_, and α_4_ as well as for the WF motifs W1, W2, W5, and W6. Unfortunately, the lack of chemical shift assignments prevents conclusions for W3, W4 and the transient helix α_1_. Interestingly, the helical content of the W0 motif, which lacks a tryptophan, is not much affected by bicelle binding.

**FIGURE 2:**
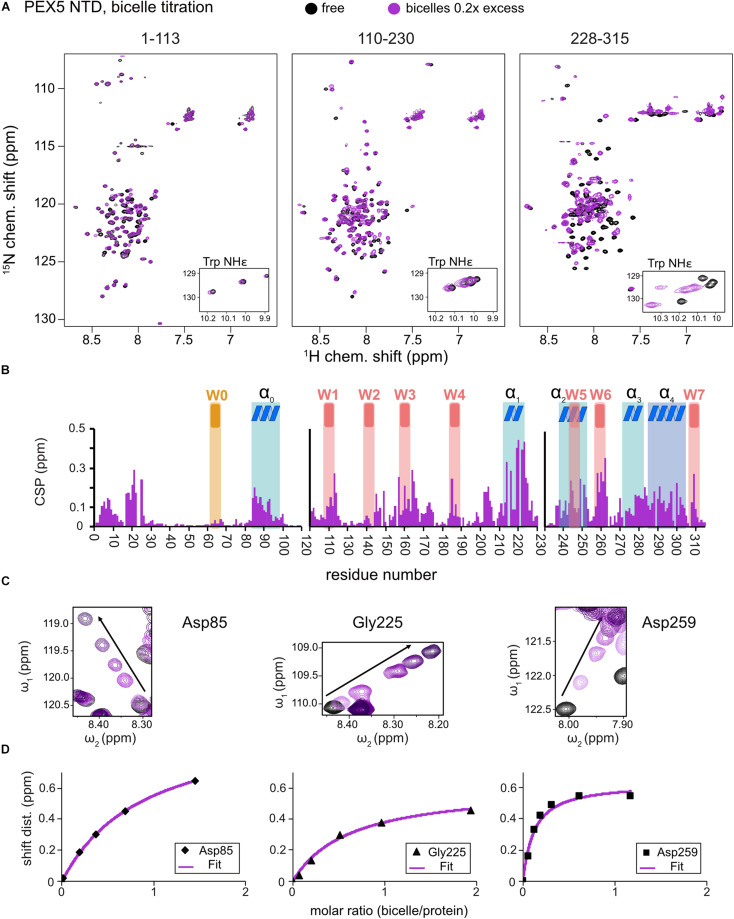
PEX5 NTD – membrane binding. ^1^H,^15^N HSQC NMR spectra overlay **(A)** of free PEX5 1–113, 110–230, and 228–315 (back) and in the presence of 0.2x molar excess of DMPC/D7PC bicelles with a *q-*value of 0.2. The subconstruct 228–315 is largely affected by 0.2x molar excess while the constructs 1–113 and 110–230 show comparable effects at a molar excess of 0.7x and 0.9x **(B)**. Tracing the chemical shift perturbations of largely affected residues Asp85, Gly225, and Asp259 of the subconstructs **(C)** and plotting shift distance against the molar ratio of bicelle to protein **(D)** Fitting of the NMR titration data (chemical shift difference to the free state) to a one-site binding model as a function of the molar bicelle:protein ratio (see section “Materials and Methods”).

Large chemical shift perturbations (CSP) are seen for the last third of PEX5 NTD (Figures 1E, 2A). The first 200 residues experience significant spectral changes only at equimolar protein/bicelle ratio, i.e., 5- fold higher bicelle concentration (Figure 2B). The strongest CSPs are mostly found in helical regions, including some of the WF-like motifs, suggesting these regions mediate the membrane interaction (Figures 1E, 2B). The C-terminal region of the PEX5 NTD, encompassing W5, W6, and W7, shows the largest CSPs, suggesting the strongest membrane interaction compared to the other regions. This is reflected in the membrane affinities derived from the NMR titrations. The apparent dissociation constants for the bicelle interactions are *K*_*D*_^*app*^ = 196 ± 16 μM, 82 ± 13 μM, and 20 ± 7 μM, for the regions comprising PEX5 residues 1–113, 110–230, and 228–315, respectively (Table 1 and Figures 2C,D). Interestingly, a common feature of the helical regions involved in membrane binding is the presence of one or more phenylalanine and or tryptophan residues (Figure 1F).

**TABLE 1:** NMR-derived membrane binding affinities.

Protein	*K*_*D*_ DMPC (μM)	K_*D*_^*app*^ bicelle (μM)
PEX5 1–113	9040 ± 746	196 ± 16
PEX5 110–230	3763 ± 589	82 ± 13
PEX5 228–315	916 ± 333	20 ± 7
PEX14 16–80	79 ± 13	1.7 ± 0.3

Taken together the NMR data reveal an unexpected extent of regions adopting an amphipathic α-helical conformation in the PEX5 NTD, which are stabilized or induced by membrane interactions.

### PEX14 NTD Interaction With Membranes

**FIGURE 3:**
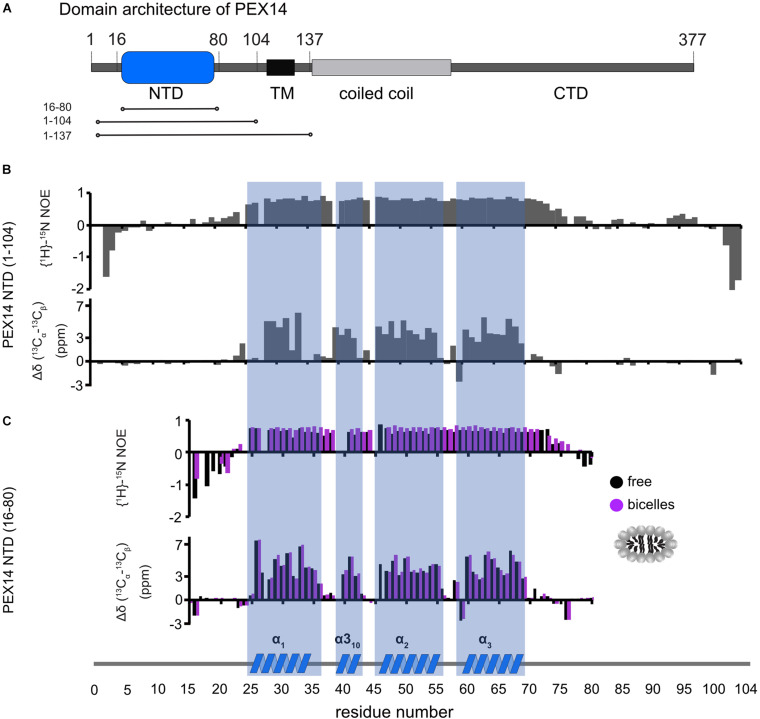
Human PEX14 NTD secondary structure. **(A)** Human PEX14 comprises a globular N-terminal domain (NTD) and a short transmembrane span which is followed by a coiled coil region and an unstructured C-terminal domain. **(B,C)** {^1^H}-^15^N heteronuclear NOE (top) and ^13^C secondary chemical shifts (bottom) of **(B)** the PEX14 NTD (residues 1–104) free in solution and **(C)** of the globular domain (residues 16–80) in solution (black boxes) and in the presence of bicelles (magenta boxes).

**FIGURE 4:**
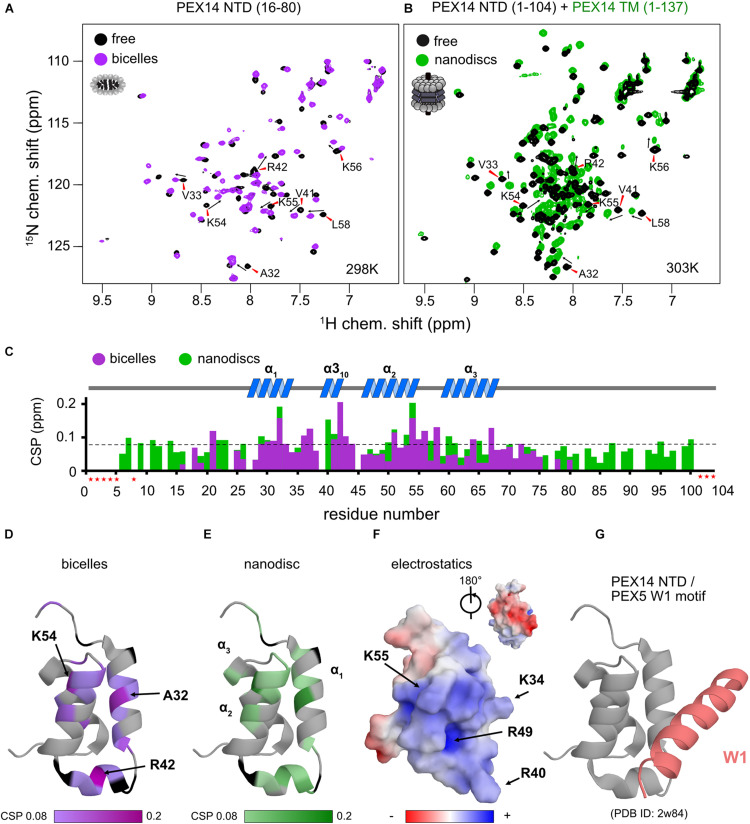
The PEX14 NTD interacts with bicelles and nanodiscs. **(A)** ^1^H,^15^N HSQC NMR spectra of the isolated NTD (16–80) in the absence (black) and presence of bicelles (1.1-fold molar excess, magenta). **(B)** Spectra of the extended PEX14 NTD (residues 1–104) in solution with the PEX14 TM (residues 1–137), anchored in nanodiscs. **(C)** Chemical shift perturbations vs. residue in the presence of bicelles (magenta) and nanodiscs (green). **(D)** Mapping of the CSPs shown in **(C)** in the presence of bicelles **(D)** and anchored to nanodiscs **(E)** onto the structure of the globular domain in the PEX14 NTD. Hotspots cluster around Ala32, Arg42, and Lys54. **(F)** The membrane binding interface is strongly positive charged due to the presence of Arg and Lys residues, as seen by electrostatic surface rendering. **(G)** The membrane binding surface partially overlaps with binding site of the PEX14 NTD with WxxxF/H motifs from the PEX5 NTD.

PEX14 is embedded in the peroxisomal membrane by a transmembrane region predicted for residues 107–129. Given that the PEX14 NTD is in close proximity to this transmembrane region (Figure 3A), we wondered whether the NTD also has some intrinsic affinity to the membrane in the absence of the transmembrane region and if this could affect the interactions with PEX5. We first confirmed that the N-terminal region of PEX14 up to the transmembrane span (residues 1–104) harbors the α-helical globular domain (residues 16–80) but is otherwise unstructured (Figure 3B). This is indeed demonstrated by the virtually identical secondary chemical shifts for the region comprising the globular domain, while the flanking regions exhibit random coil chemical shifts and low heteronuclear NOE values and are thus intrinsically disordered (Figures 3B,C). The heteronuclear NOE data show a small increased rigidity for residues 90–97 (corresponding to the amino acid sequence QPPHLISQP), which is often observed in P-rich regions (Chen et al., 2013). We therefore studied the potential membrane interaction of the PEX14 NTD focusing on the globular domain (residues 16–80). For this, we performed NMR titration experiments of a ^15^N-labeled sample of the globular α-helical fold in the PEX14 NTD (residues 16–80) (Neufeld et al., 2009) with preformed bicelles up to full saturation (Figure 4A and Supplementary Figures 3A,B). Substantial chemical shift perturbations and some line-broadening are observed for many residues indicating a significant interaction of the PEX14 NTD with the bicellar surface. The secondary chemical shifts and heteronuclear NOE values in the absence and presence of bicelles are very similar (Figure 3C) and the overall spectral dispersion of the NMR signals is not affected (Figure 4A), demonstrating that the globular fold of the domain is still intact. This is also supported by the very similar circular dichroism spectra and melting temperatures in the absence and presence of bicelles (Supplementary Figures 3C,D). To confirm the significance of the spectral changes in the presence of bicelles we performed a control experiment with an unrelated RNA binding domain, which is expected to not interact with membranes. Here, virtually no spectral changes are seen upon addition of bicelles (Supplementary Figure 3E).

We next wanted to explore whether the membrane anchoring of the PEX14 NTD affects the membrane interaction of the globular domain. For this we compared NMR spectra of the PEX14 NTD (1–104) with a construct that additionally includes the transmembrane region, PEX14 TM (residues 1–137), which was assembled into nanodiscs (Figure 4B). Notably, mapping the CSPs onto the sequence of PEX14 reveals very similar chemical shift perturbations (Figure 4C). This shows that the PEX14 NTD has an intrinsic membrane affinity, that is independent of being anchored to the membrane via the TM region. The strongest CSPs (above a threshold of 0.08 ppm) are seen in α-helices 1 and 2 and the α3_10_ helix (Figure 4C). Mapping the CSPs onto the structure of the PEX14 NTD (Figures 4D,E) reveals three hotspots for the interaction, located around Ala32 (helix α1), Arg42 (α3_10_), and Lys54 (helix α2). Arginine and lysine residues in these three helices form a positively charged surface while the other side of the domain is mainly negatively charged (Figure 4F). Interestingly, the positively charged surface partially overlaps with the binding interface for WF motifs (Figure 4G), where PEX14 Lys56 is reported to form an important salt bridge with PEX5 Glu121 (Neufeld et al., 2009). This suggests an at least partially competitive binding of PEX14 NTD to membranes and WF-like motifs in PEX5.

### PEX5 PEX14 Interaction in the Presence of Lipids

**FIGURE 5:**
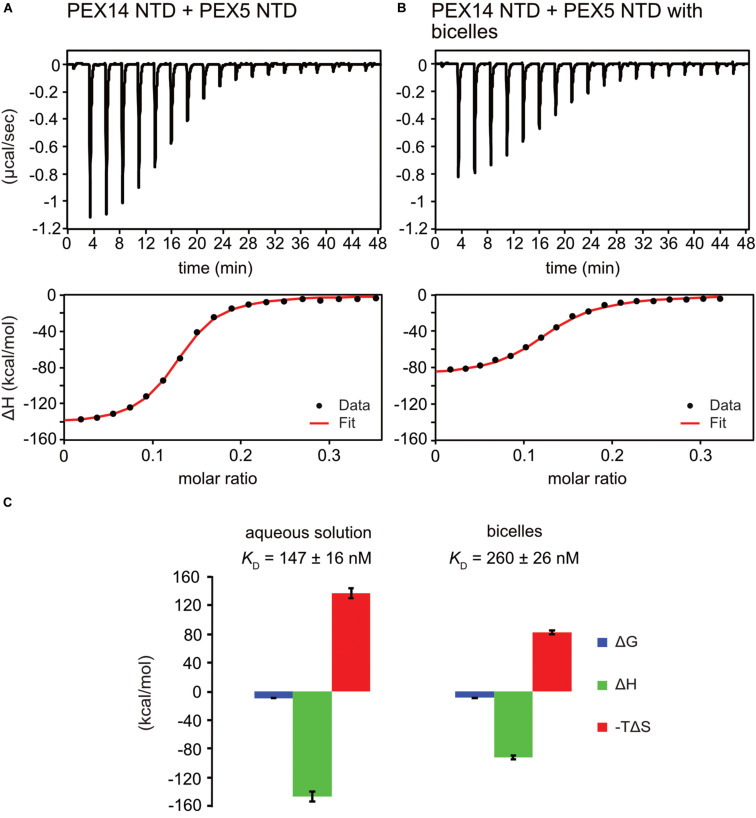
PEX5 - PEX14 interaction by isothermal titration calorimetry. ITC experiments were performed as triplicates. **(A)** Titration of 50 μM PEX5 in aqueous buffer into a 30 μM aqueous solution of PEX14. **(B)** The same experiment in the presence of 44 μM bicelle corresponding to full saturation of PEX5 and PEX14 with 0.9- fold and 1.5- fold molar excess, respectively. **(C)** The titration experiments in membrane-like environment show reduced binding enthalpies ΔH and entropic contribution –TΔS and slightly reduced affinity compared to experiments in aqueous solution.

To investigate the potential competitive binding of PEX14 NTD to membranes and WF-like motifs in PEX5, we performed isothermal titration calorimetry (ITC) with the PEX5 NTD (residues 1–315) and PEX14 NTD (1–104) in aqueous solution (Figure 5A and Supplementary Figure 4A) or in the presence of bicelles (Figure 5B and Supplementary Figure 4B). The bicelle concentration used correspond to a 0.9-fold and 1.5-fold molar excess for PEX14 and PEX5, respectively, to ensure saturated membrane binding. Since PEX5 NTD harbors eight possible binding sites for PEX14, the number of sites was fitted to 1/8. This results in a dissociation constant, *K*_*D*_ of 147 ± 16 nM, for the interaction of the PEX5 and PEX14 NTDs in aqueous solution (Figure 5C and Table 2). The free Gibb’s energy ΔG = −9.3 kcal/mol is composed of a binding enthalpy, ΔH = −147.0 kcal/mol and –TΔS = 137.7 kcal/mol, indicating that the interaction is enthalpy driven with a negative change of entropy ΔS. In the presence of bicelles *K*_*D*_ = 260 ± 26 nM, with ΔG = −8.9 kcal/mol, and thus in the same range as without bicelles. Interestingly, both the binding enthalpy and the entropic contributions are reduced by about 60% to ΔH = −92.0 kcal/mol and –TΔS = 83.0 kcal/mol, respectively, compared to the interaction between the two proteins in the absence of membrane. The reduced binding enthalpy is consistent with a partially competitive binding between the two proteins toward each other and the bicelles. However, the enthalpy reduction is compensated by a reduced entropy loss associated with the PEX5-PEX14 interaction in the presence of a membrane environment. This may result from the increased helical conformation observed for WF motifs in the presence of membrane, which thereby can reduce the entropic cost associated with the formation of a full helical conformation when bound to the PEX14 NTD.

**TABLE 2:** ITC titration with PEX5 NTD (1–315) on PEX14 NTD (1–104).

Buffer conditions	*N*	*K*_*D*_ (nM)	ΔG (kcal/mol)	ΔH (kcal/mol)	−TΔS (kcal/mol)
Buffer	0.125	147 ± 16	−9.3 ± 0.07	−147.0 ± 7	137.7 ± 7
Bicelles	0.125	260 ± 26	−8.9 ± 0.06	−92.0 ± 3	83.0 ± 3

## Discussion

Here we present a comprehensive NMR and biophysical analysis of the membrane-associated N-terminal regions of the peroxisomal targeting receptor PEX5 and its binding partner PEX14, which play critical roles for initial steps of the assembly of the peroxisome translocon.

The PEX5 NTD contains eight WF-like binding motifs (W0-W7) that are recognized by the PEX14 NTD. Although this region is overall largely disordered our study reveals that some of the WF-motifs exhibit partial helical conformation, with the highest propensity for W5. The preformed helical conformation may reduce the entropic loss associated with the formation of helix upon complex formation with PEX14. This is consistent with the fact that the W5 motif has the highest binding affinity amongst the eight WF-like binding motifs (Gopalswamy et al., in preparation). Surprisingly, we identified five conserved, amphipathic helical regions α_0_ (residues 81–96), α_1_ (210–220), α_2_ (237–250), α_3_ (271–285), and α_4_ (287–301), where helices α_0_ to α_3_ are transient and partially formed, while helix α_4_ seems almost fully formed in solution (Figure 1C). Interaction with membrane-mimicking bicelles stabilizes the α-helical character of these helices including the WF-motifs, with the exception of the W0 motif, which lacks a tryptophan as second aromatic residue. The stabilization of the amphipathic helices might rise from electrostatic interactions from charged amino acids which are often present on the hydrophilic surface (Giménez-Andrés et al., 2018), as especially seen for helices α_3_ and α_4_ of PEX5 (Figure 1F).

The NMR titrations with bicelles revealed that residues 210–310 in the PEX5 NTD represent the most important interaction site for membrane binding (Figure 1E). This region harbors the helices α_1_ to α_4_, which share as a common feature the exposure of phenylalanine and/or tryptophan side chains on the hydrophobic face of the amphipathic helix (Figure 1F). Interestingly, helix α_0_, which lacks an aromatic residue on its hydrophobic face does not show significant spectral changes upon bicelle binding. This suggests that the aromatic residues are important as anchors for the membrane interaction, as is commonly observed for amphipathic helices (Cornell and Taneva, 2006; de Jesus and Allen, 2013; Giménez-Andrés et al., 2018). Amphipathic helices found in the PEX5 NTD are 10 to 15 residues in length exposing four to seven aliphatic residues, which corresponds to two to four helical turns. Such short helices can be found in membrane channels, while the average length helices in *bona fide* transmembrane proteins (TMPs) is 17.3 residues with a length of about 26 Å (Hildebrand et al., 2004). Our NMR titrations show significant chemical shift perturbations in the presence of bicelles (Figure 2), consistent with a micromolar binding to the bicelle surface. Note, that this experimental setup does not allow us to make conclusions about a potential transmembrane spanning of these regions by PEX5. Thus, we conclude that the PEX5 NTD has significant affinity to the membrane, which may play a role in the formation of the peroxisome translocon.

Our NMR data show that the secondary structure and overall fold of the PEX14 NTD (16–80) is not altered in the presence of membrane-mimicking bicelles (Figure 3C and Supplementary Figures 3C,D). Nevertheless, significant CSPs are observed upon bicelle binding for amide signals in helices α1 and α2 and the short helix α3_10_ (Ala32, Arg42, and Lys54), which highlights this region as membrane interaction surface (Figures 4C,D). Notably, NMR spectral changes seen for the PEX14 NTD when inserted into a phospholipid nanodisc by the presence of the transmembrane region, identify the same membrane interaction surface of the globular helical domain in the PEX14 NTD (Figure 4E). Additional line broadening is seen for some of the lysine and arginine residues in the binding surface presumably reflecting a stronger interaction due to the anchoring of the protein in the nanodisc. The NMR data demonstrate that the small helical fold in the PEX14 NTD has an intrinsic although weak affinity to the membrane surface, independent of the presence of the membrane-spanning helix. The membrane binding helices represent a positively charged surface (Figure 4F) harboring numerous Arg and Lys residues.

Surprisingly, the PEX14 NTD membrane interaction surface partially overlaps with the interface for the bi-aromatic WF-motifs in the PEX5 NTD (Figures 4D–G). This suggests at least a partial competition in the binding to the PEX14 NTD. Indeed, our ITC data for the PEX14/PEX5 interaction in the absence and presence of bicelles show a minor decrease of affinity from 150 to 250 nM in the presence bicelles, respectively (Figure 5 and Table 2). In both titration series, we observe negative binding enthalpy and entropy, which demonstrates that the interaction is driven by enthalpy. The negative entropy can be explained by the loss of conformational flexibility upon formation of an α-helical conformation of the WF peptides upon binding to the PEX14 NTD. Interestingly, in the presence of bicelles both ΔH and –TΔS are reduced by ≈60%. This is consistent with a competitive binding of the PEX5 WF-motif and the bicelle to the PEX14 NTD. However, the free binding enthalpy remains very similar as a result of enthalpy/entropy compensation. The partial competition for the PEX14 binding surface reduces the binding enthalpy ΔH, but is likely compensated by the fact that both binding partners are preferentially localize at the membrane and that the membrane interaction increases the pre-existing helical conformation of the WF-motifs, such that loss of conformational entropy from disordered to helical conformation of the WF peptides is reduced, compared to an interaction in the absence of membrane-mimicking bicelles.

## Conclusion

We show that the PEX5 NTD, while being overall unstructured, exhibits a number of weakly populated, transient helical regions, which have amphipathic character. Notably the helical propensity is stabilized by a weak micromolar interaction with the membrane (Figure 2). As judged from the NMR chemical shift perturbations the largest contribution to the membrane binding can be mapped to residues 210–310 in the PEX5 NTD, which comprises the two WF motifs W5 and W6. The other WF motifs and the pre-existing helix α_0_ which lacks an aromatic residue, are much less affected in the presence of the membrane. This supports the hypothesis that the WF-like motifs in the N-terminal region of the PEX5 NTD can initiate PEX14 binding (Neuhaus et al., 2014), while the region comprising residues 210–310 help to stabilize PEX5 at the membrane. The PEX14 NTD itself is weakly membrane-associated with the membrane with micromolar affinity (Supplementary Figure 3), but this interaction is readily competed out by the PEX5 WF-like motifs, which bind with significantly stronger binding affinity. The weak membrane interactions of the PEX14 NTDs may provide a proof-reading mechanism to avoid random binding events with unspecific targets from the cytosolic compartment.

## Materials and Methods

### Molecular Cloning

The full length genes of human PEX5 (UniProtKB no. P50542) and human PEX14 (UniProtKB no. O75381) were optimized according to the codon usage of *Escherichia coli* and synthesized by IDT (IDT Europe GmbH, Germany). These sequences were used as templates to generate PEX5 (1–113), PEX5 (110–230), PEX5 (228–315), PEX5 (1–315), PEX14 (1–104), and PEX14 (1–137) constructs by polymerase chain reaction (PCR) amplification using the following primers:

PEX5        1–110:      F:                              aaaccatggcgatgcgcgaac R: aaaggtaccttacgccagatcggcaacacc
PEX5     110–230     F:                      aaaccatggccgatctggcgttatcg R: aaaggtaccttactctaaactgacctggccttc
PEX5     228–315     F:      aaaccatggagagtttagagtctggtgccggatc R: aaaggtaccttagaggtcatcatag
PEX5         1–335      F:            gatcccatggcaatgcgggagctggtggag R: gatcgcggccgctagtgatcagccaaggggttctcc
PEX14       1–104      F:                       aaaccatggctagcagcgaacagg R: aaaggtaccttaactacccgccggagaatacg
PEX14        1–137      F:                      aaaccatggctagcagcgaacagg R: aaaggtaccttaacctaagatcagcggaaggagg

where F/R refers to forward and reverse primers, respectively.

PEX5 fragments PEX5 (1–113), PEX5 (110–230), PEX5 (228–315), and PEX5 (1–315) were cloned into the bacterial expression pETM10 vector with a non-cleavable N-terminal His_6_-tag and PEX14 fragments was cloned into pETM11 vector with His_6_-tagged followed by a tobacco etch virus (TEV) cleavage site (EMBL, G. Stier) using *Nco*I and *Kpn*I restriction sites. PEX5 (1–335) was PCR-amplified using pET9d-His-TEV-PEX5L (Schliebs et al., 1999) as a template and subcloned into *Nco*I/*Not*I-digested pET9d expression plasmid.

### Protein Sample Preparation

PEX constructs were transformed into *E. coli* BL21 (DE3) cells and expressed in LB or isotope-enriched M9 minimal medium. Single colonies were picked randomly and cultured in the medium with 50 μg/ml kanamycin overnight at 37°C. Overnight cultures were grown at 37°C, diluted 50-fold, and grown up to an optical density of 0.4–0.6 at 600 nm. Protein expression was induced at 37°C with 0.1 mM IPTG for PEX14 (1–104), 0.5 mM IPTG for PEX14 (1–137), and 1 mM IPTG for PEX5 constructs. While PEX5 and PEX14 (1–104) were expressed for 18 h at 18°C, PEX14 (1–137) remained for 4 h at 37°C before being harvested. The cells were harvested by centrifugation at 5,000 rpm for 20 min at 4°C. For protein purification the cell pellets were resuspended in the different binding buffers described below and lysed by pulsed sonication (5 min, 40% power, large probe, Fisher Scientific model 550) followed by centrifugation at 15,000 rpm for 1 h. All proteins were purified using gravity flow Ni-NTA (Qiagen, Monheim am Rhein, Germany) affinity chromatography which can be described in three steps. First a binding step where the supernatant of the cell lysate is applied to the column. Second, a wash step where endogenous proteins where removed and a third step where the protein of interest is eluted from the column. However, the different natures of the PEX5/PEX14 constructs bring the need of different buffer compositions.

Intrinsically disordered PEX5 protein constructs were lysed in buffer containing 50 mM sodium phosphate (NaP) buffer, pH 8, 300 mM NaCl, 10 mM imidazole, and 8 M Urea. The urea denatures all proteins and prevents binding of contaminants. After binding to the column, urea was removed in an extensive washing step using urea-free buffer. Then, the proteins were eluted in a high imidazole buffer (50 mM NaP, pH 8, 300 mM NaCl, 500 mM imidazole) before final purification via size exclusion chromatography (Superdex S75, 16/600, GE Healthcare, Rosenberg, Sweden) in NMR buffer (see below) and lyophilized for long term storage.

The PEX14 constructs comprising residues 1–104 and 1–137 were lysed in 50 mM Tris/HCl, pH 8.0, 100 mM NaCl, and protease inhibitor mix (Serva, Heidelberg, Germany). After lysis, the PEX14 1–137 suspension was adjusted to 1 M NaCl and 1% (w/v) dodecylphosphocholine (DPC). After binding to the column a wash step with additional 20 mM imidazole was performed. While salt concentration was kept constant for PEX14 1–104, NaCl and DPC concentrations for PEX14 1–137 were lowered to 500 mM and 0.2% (w/v), respectively. The protein was eluted by increasing imidazole to 500 mM. PEX14 1–137 was subsequently further purified for nanodisc assembly including a buffer exchange to 50 mM Tris, pH 8.0, 100 mM NaCl, 0.1% (w/v) DPC using a Superdex S200 (16/600, GE Healthcare). PEX14 (1–104) was final purified after TEV cleavage running a reverse Ni^2+^ column and a Superdex S75 (16/600), where the buffer was changed to NMR buffer containing 50 mM NaP pH 6.5 and 100 mM NaCl. The PEX14 16–80W construct (with a C-terminal Trp) was expressed and purified as described previously (Neufeld et al., 2009). Protein expression and purification of full-length PEX5 and PEX5 (1–335) was carried out as described (Schliebs et al., 1999). Protein purification and quality was confirmed by SDS PAGE (Supplementary Figures 5A,B,D).

Uniformly ^15^N or ^15^N, ^13^C labeled proteins were expressed in H_2_O or D_2_O M9 minimal medium supplemented with 50 μg/ml kanamycin, 1 g/liter ^15^N NH_4_Cl and 2 g/liter hydrated or deuterated [U-^13^C] glucose as the sole sources of nitrogen and carbon, respectively.

### Bicelles and Nanodiscs Preparation

Lipids 1,2-Diheptanoyl-*sn*-glycero-3-phosphocholine (D7PC) and 1,2-Dimyristoyl-*sn*-glycero-3-phosphocholine (DMPC) were purchased from Avanti Polar Lipids (Alabaster, United States). Bicelles were prepared according to the established protocols (Sommer et al., 2013). Briefly, water free D7PC and DMPC were dissolved in chloroform to generate stock solutions of 500 mM and 100 mM, respectively. The lipids were mixed in a ratio of 1 to 1, dried under vacuum and rehydrated in 20 mM NaP, pH 6.5, 100 mM NaCl, 0.02% (w/v) NaN_3_ to generate a 240 mM lipid stock. The bicelles with *q* = 0.2 were formed by several freeze and thaw cycles in liquid N_2_ yielding a clear, viscous bicelle solution of 870 μM concentration. The bicelle concentration was calculated based on the number of DMPC molecules in one bicelle. The radius *R* of the bilayer region of the bicelle (for *q* = 0.2) was calculated to be 2.04 nm using the formula *R* = 1/2*r*q[π + (π^2^ + 8/q)^1/2^] assuming a bilayer thickness of 4 nm with a radius *r* = 2 nm (Klopfer and Hagn, 2019). Thus the calculated surface area of the bicelle is 1307 Å^2^, which corresponds to 46 DMPC molecules (given a surface area of 57 Å^2^ per DMPC molecule).

Nanodisc assembly with freshly purified PEX14 (1–137) was performed with a lipid mixture of 75% deuterated DMPC, 25% deuterated DMPG (FB Reagents, Cambridge, United States) and the 19.5 kDa scaffold protein MSP1D1Δ5 as described (Hagn et al., 2018). Buffer exchange to NMR buffer was done via size exclusion chromatography on a Superdex S200 (16/600). To confirm successful reconstitution of PEX14 (1–137) peak fractions were analyzed by SDS-PAGE (Supplementary Figure 5C). Protein concentration of 150 mM of 250 μl sample was then transferred to a Shigemi (Shigemi Inc., Allison Park, United States) tube for NMR experiments.

### Liposome Preparation and Flotation Assay

1,2-dioleoyl-*sn*-glycero-3-phosphocholine (DOPC) and 1,2-dioleoyl-*sn*-glycero-3-phosphoethanolamine (DOPE) were purchased from Avanti Polar Lipids, Inc. (United States). DOPC/DOPE lipids were mixed with a ratio of 7 to 3 in 50 mM NaCl, 20 mM Tris, pH 7.4 as described (Kerssen et al., 2006). In addition, 10 mM DPMC, purchased from Sigma-Aldrich (Germany), was resuspended in 50 mM NaCl, 20 mM Tris, pH 7.4. Small unilamellar vesicles (SUVs) were obtained by sonication of the multilamellar vesicle (MLV) suspension using an ultrasonic bath (Sonorex RK 52, Bandelin) followed by 10 cycles of freezing and thawing (Pick, 1981).

Liposomes, either DOPC/DOPE or DMPC, were incubated with 1.5 nmol purified human PEX5 or PEX5 (1–335) for 1 h at room temperature (ratio protein/lipid: 1/750). Incubation of liposomes with protein and all following steps were performed in 50 mM NaCl, 20 mM Tris–HCl, pH 7.4. The loading samples were adjusted to a sucrose concentration of 45% (w/v) using 65% (w/v) sucrose solution and 0.4 ml of each sample were layered onto 0.52 ml 50% sucrose cushion at the bottom of 11 ml ultracentrifuge tubes. 1.3 ml 40% (w/v) sucrose, 5.1 ml 25% (w/v) sucrose 2.6 ml of buffer without sucrose were stepwise added. After ultracentrifugation for 4 h at 175,000 × *g* at 4°C in a swing-out rotor, the linear gradient (0 to 50% (w/v) sucrose) was collected as ten 1 ml fractions from top to bottom. The fractions were separated by SDS-PAGE and analyzed by immunoblotting using polyclonal rabbit anti PEX5 antibodies.

### NMR Spectroscopy

NMR data were collected on Bruker Avance III spectrometers operating at 500, 600, 800, 900 or 950 MHz, equipped with cryogenic probes. The sequential assignment of backbone resonances for PEX5 fragments and PEX14 (1–104) were performed based on heteronuclear experiments such as ^1^H-^15^N-HSQC, HNCA, HN(CO)CA, CBCA(CO)NH, HNCACB, HNCO, HN(CA)CO, HN(CA)NNH and H(NCA)NNH (Weisemann et al., 1993; Sattler et al., 1999). {^1^H}-^15^N heteronuclear NOE (hetNOE) experiments (Farrow et al., 1994) were performed using the pulse sequence hsqcnoef3gpsi (Bruker, Avance version 12.01.11) with a 5 s interscan delay. NOE values are given simply by the ratio of the peak heights in the experiment with and without proton saturation (hetNOE = I_*sat*_/I_0_) (Renner et al., 2002). NMR-Spectra were processed using Topspin (Bruker Biospin, Rheinstetten, Germany) or NMRPipe (Delaglio et al., 1995) and analyzed using CcpNMR Analysis 2.4.2 (Vranken et al., 2005).

All NMR experiments with PEX5 were recorded in 20 mM NaP pH 6.5, 100 mM NaCl, 0.02% (v/v) NaN_3_, and 2 mM DTT at 298°K at 600 MHz for triple resonance experiments or at 500 MHz for hetNOE experiments. Triple resonance experiments of free PEX5 1–113, 110–230, and 228–315 were performed at concentrations of 225 μM, 200 μM, and 200 μM, respectively. Assignment experiments for PEX5 1–113, 110–230, and 228–315 in the presence of 870 μM bicelles (*q* = 0.2) were recorded at 350 μM to 430 μM. {^1^H}-^15^N heteronuclear NOE experiments were performed by dissolving lyophilized ^15^N-labeled PEX5 1–113, PEX5 110–230 and PEX5 228–315 in a buffer containing 20 mM NaP pH 6.5, 100 mM NaCl, 2 mM DTT to a final concentration of 190 μM, 90 μM, and 165 μM, respectively.

Experiments with all PEX14 constructs were recorded in 20 mM NaP pH 6.5, 100 mM NaCl. While spectra of PEX14 (16-80W) were recorded at 298°K at 600 MHz proton Larmor frequency, NMR experiments for PEX14 1–104 and 1–137 without and with nanodiscs were collected at 298°K or 303°K at 800, 900, or 950 MHz proton Larmor frequency, respectively. Backbone assignments and hetNOE experiments of PEX14 16–80W in the presence of bicelles were recorded at 950 μM in 150 μM bicelle solution. Triple resonance experiments for PEX14 1–104 were recorded on 950 MHz at a concentration of 750 μM and 298°K. Nanodiscs of 6 nm size with PEX14 1–137 were assembled and purified as described above, 2D ^1^H-^15^N HSQC experiments were measured at a final concentration of 150 μM at 303°K and 900 MHz.

Titration experiments of preformed bicelles to ^15^N-labeled PEX5 1–113, 110–230, and 228–315 at 280 μM, 150 μM, and 150 μM and PEX14 16-80W at 110 μM were performed with increasing bicelle concentration up to 1.5-fold, 2-fold, and 1-fold molar excess, respectively. The chemical shift perturbation (Δδ*_*avg*_*) was calculated by using formula Δδ*_*avg*_* = [(Δδ*_*H*_*)^2^ + (Δδ*_*N*_*/6.3)^2^]^0.5^. Dissociation constants (*K*_*D*_) were fitted to DMPC concentration with a one-site specific binding model within Origin software (OriginLab Corporation, United States). The equation used for the fitting is Δδ = Δδ*_*max*_*/(2 [P_*t*_])*{[L] + [P_*t*_] + *K*_*D*_ – (([L] + [P_*t*_] + *K*_*D*_)^2^-4[P_*t*_][L])^1/2^}, where Δδ is the individual and Δδ*_*max*_* the maximum shift distance, P_*t*_ is the total protein concentration and [L] the DMPC ligand concentration. The *K*_*D*_ of ∼20 representative residues of each construct was fitted to the DMPC concentration assuming that all DMPC molecules are associated with bicelles, whereas partially water soluble D7PC molecules may exist in an equilibrium between solution and bicelle-bound. Bicelle concentrations were derived from the DPMC concentration by scaling with a factor of 1/46 (according to the number of DPMC molecules per bicelle, see above) to obtain apparent dissociation constants *K*_*D*_^*app*^ for the bicelle interaction.

### Isothermal Titration Calorimetry (ITC)

Isothermal titration calorimetry (ITC) measurements were performed as triplicates at 25°C using a MicroCal PEAQ-ITC (Malvern Instruments Ltd., United Kingdom) calorimeter. Buffer conditions were 20 mM NaP pH 6.5, 100 mM NaCl containing none or 44 μM bicelles. Pex5 (1–315) at a concentration of 50 μM was injected in the cell containing Pex14 (1–104) at a concentration of 30 μM. The concentration of PEX14 was corrected with the fit, since it cannot be accurate measured at 280 nm owing to the extinction coefficient of only 1490. The dilution effect of PEX5 as a control experiment was subtracted before the data were fitted to a one-site binding model using the Malvern Analysis software.

### Circular Dichroism (CD)

Far-ultraviolet circular dichroism (Far-UV CD) and thermal unfolding measurements were carried out using a Jasco J-810 spectropolarimeter equipped with a peltier thermal controller in a 0.1 cm path length quarts cuvette. Measurements were performed between 10 or 15°C to 95°C with 1°C/min scanning speed. Far UV-CD data of PEX14 (16-80W) at concentration of 25 μM in bicelle free or 44 μM bicelle (*q* = 2) in 10 mM sodium phosphate, 50 mM sodium chloride and pH 6.5 were collected at 25°C in the range of 190–260 nm wavelength. Protein-bicelle complexes were incubated for 2–3 h prior to the experiment. Spectra were collected in 10 accumulations and subtracted from the spectrum of the buffer control. Thermal unfolding spectra were collected at 222 nm. The midpoint of the folding and unfolding (*T*_*m*_) of the complex was derived from the raw data by fitting to the equations for the sigmoidal curve, Y = A2 + (A1-A2)/(1 + exp((x-x_0_)/dx)). Where A1 and A2 are the folding and unfolding intercept, respectively. x is the midpoint of the cure and dx is the slope of the curve. All curves were fitted by using Origin software (OriginLab Corporation, United States).

## Data Availability Statement

The raw data supporting the conclusions of this article will be made available by the authors, without undue reservation.

## Author Contributions

SG and MG performed and analyzed NMR and ITC experiments with bicelles. SG, MG, PZ, and ME performed cloning and protein expression and purification. ME performed NMR experiments with nanodiscs. MR, WS, and RE performed and analyzed flotation experiments. MS and MG designed the study. SG and MS wrote the manuscript. All authors contributed and approved the manuscript.

## Conflict of Interest

The authors declare that the research was conducted in the absence of any commercial or financial relationships that could be construed as a potential conflict of interest.
