# Supplementary material for: Membrane Interactions of the Peroxisomal Proteins PEX5 and PEX14
Source: Front Cell Dev Biol. 2021 Apr 16;9:651449. doi: 10.3389/fcell.2021.651449 (PMC8086558; doi:10.3389/fcell.2021.651449)
Supplement: Supplementary file 1 [file Data_Sheet_1.pdf]

Supplementary Material

Supplementary Figure 1

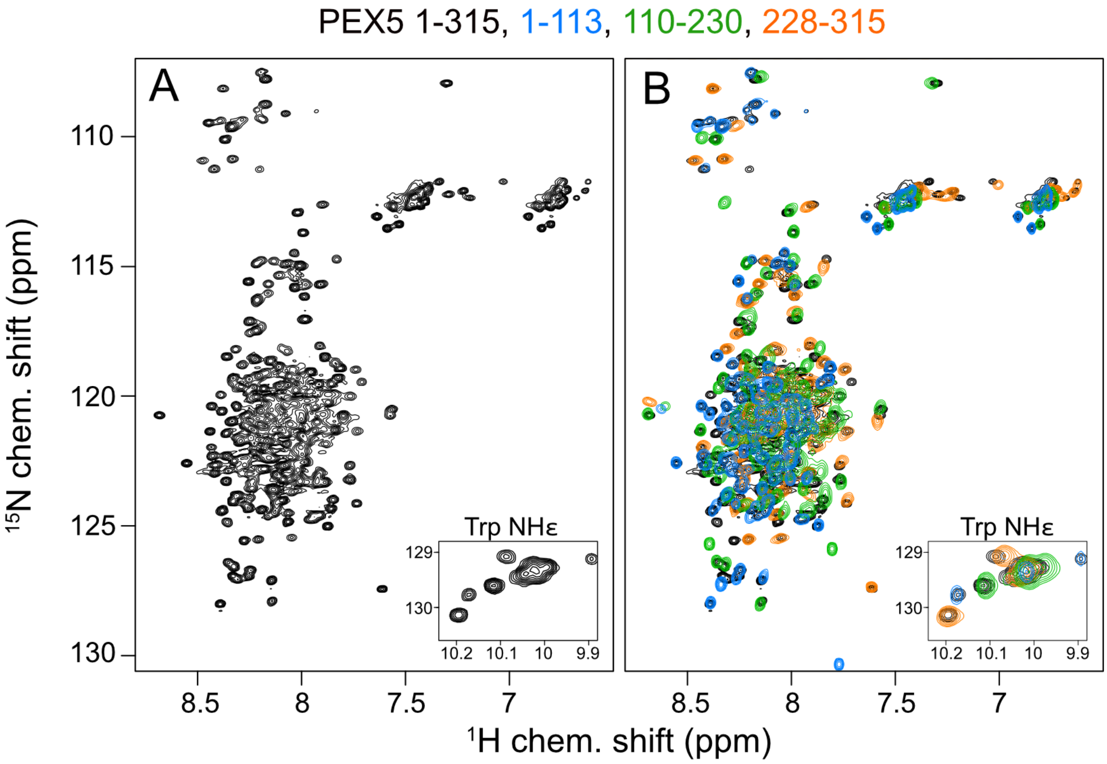

Supplementary Figure 1. ^{1}H,^{15}N HSQC NMR spectra of (A) human PEX5 NTD residues 1-315 and (B) superposition of spectra of human PEX5 residues 1-113 (blue), 110-230 (green) and 228-315 (orange) with the complete NTD (black). The spectral region showing the tryptophan side chain indole NH signals are shown as inserts.

Supplementary Figure 2

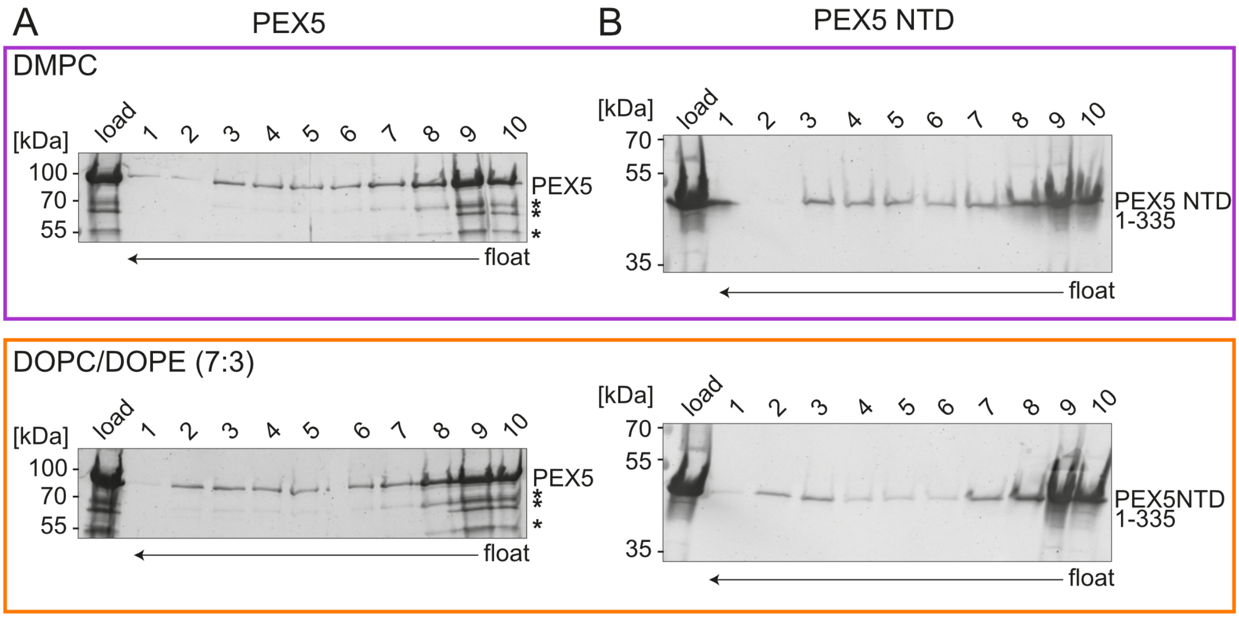

Supplementary Figure 2. Recombinant PEX5 associates with phospholipid bilayer vesicles with different composition.1.5 nmol purified human PEX5 full length (A) and PEX5 NTD (1-335) (B) were incubated for 1h at room temperature with liposomes (ratio 1:750) either consisting of DMPC (purple box) only or of a mixture of DOPC/DOPE (7:3) (orange box). The samples were subjected to flotation gradient centrifugation (50% sucrose-0% sucrose). The gradient was collected as ten fractions from top to bottom. Equal volumes of fractions were analyzed by immunoblotting using polyclonal antibodies against human Pex5L. Asterisks indicate degradation products of PEX5, which did not co-migrate with floated liposomes.

Supplementary Figure 3

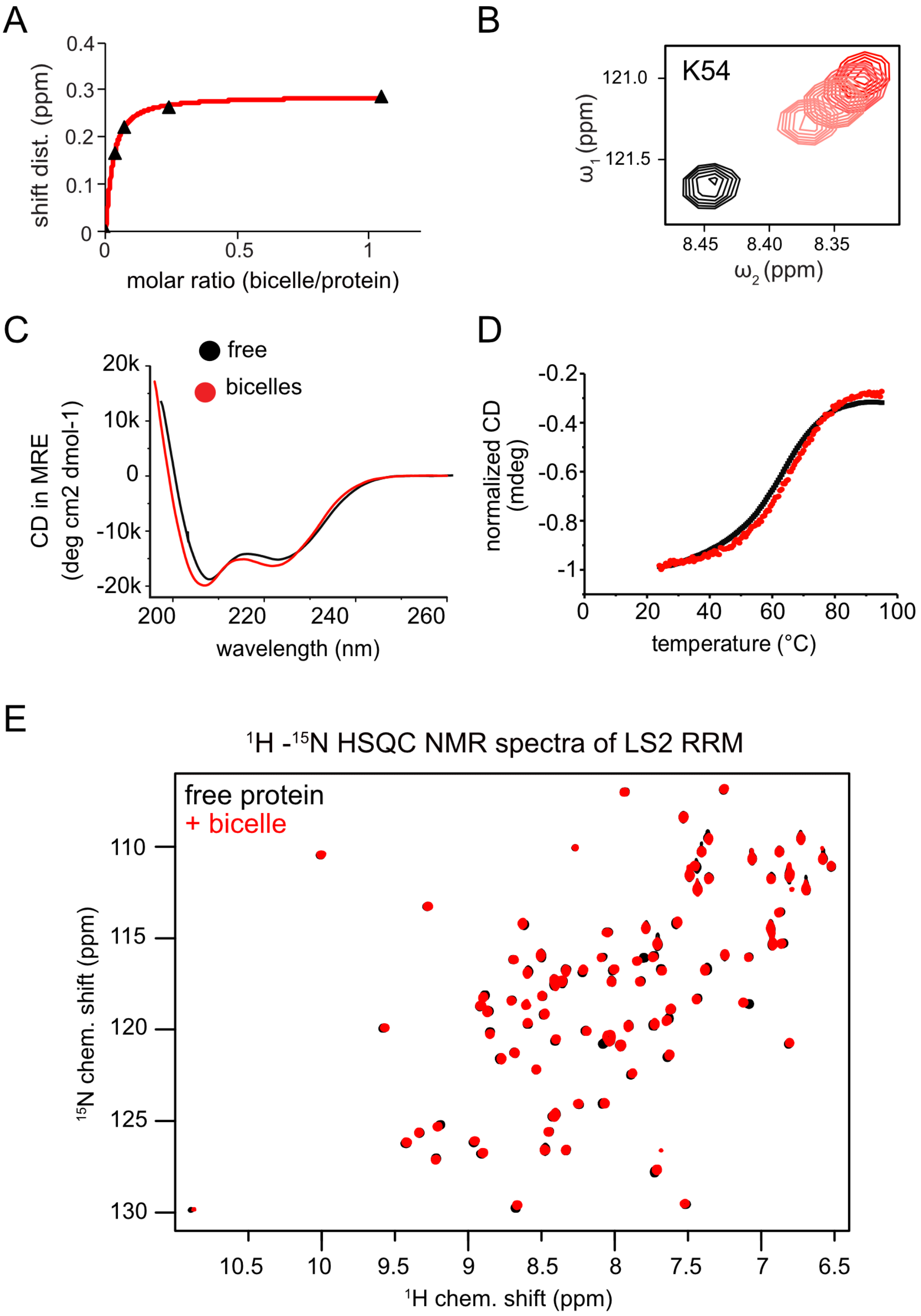

Supplementary Figure 3. Biophysical PEX14 free and in the presence of bicelles. (A) Fit of chemical shift distance vs. bicelle concentration for the amide of PEX14 K54 for K_D calculations. (B) Zoomed spectral region of ^{1}H,^{15}N HSQC NMR spectra for the amide signal of K54. (C) CD spectra of free and bicelle-bound PEX14 NTD (residues 16-80) (D) Melting curves of PEX14 NTD in buffer and in the presence of bicelles, recorded at 222nm. (E) Control titration experiment of the RRM of the *D. melanogaster* LS2 RNA binding protein with bicelles.

Supplementary Figure 4

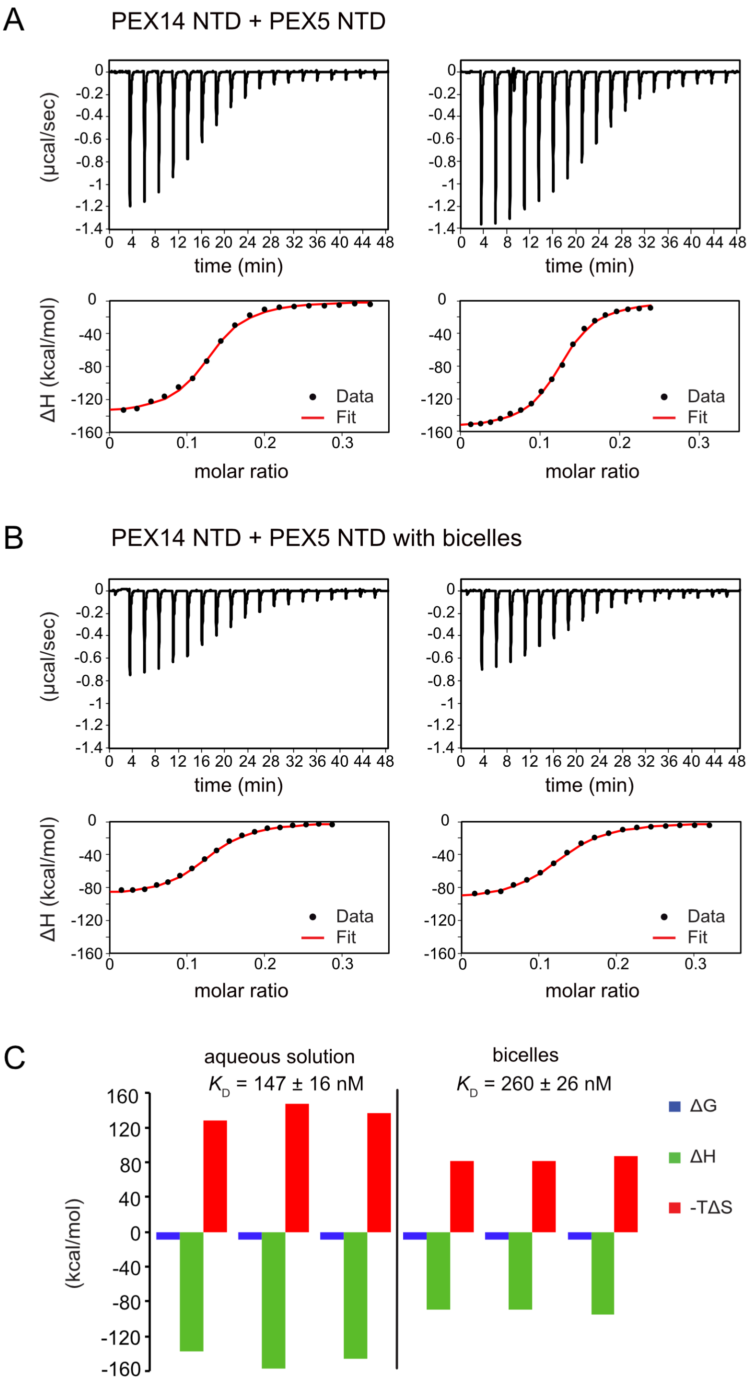

Supplementary Figure 4. ITC experiments for the PEX5 NTD and PEX14 NTD interaction. PEX5 NTD (50µM) in aqueous buffer was titrated to 30 µM PEX14 (A) in aqueous solution or (B) in bicelles containing buffer. (C) Thermodynamic parameters for the titration experiments in solution and in the presence of bicelles. The titration experiments in membrane like environment show similar Gibb's free energies ΔG (blue) but significantly reduced energies ΔH (green) and entropic contribution –TΔS (red) with a slightly reduced affinity compared to experiments in aqueous solution.

Supplementary Figure 5

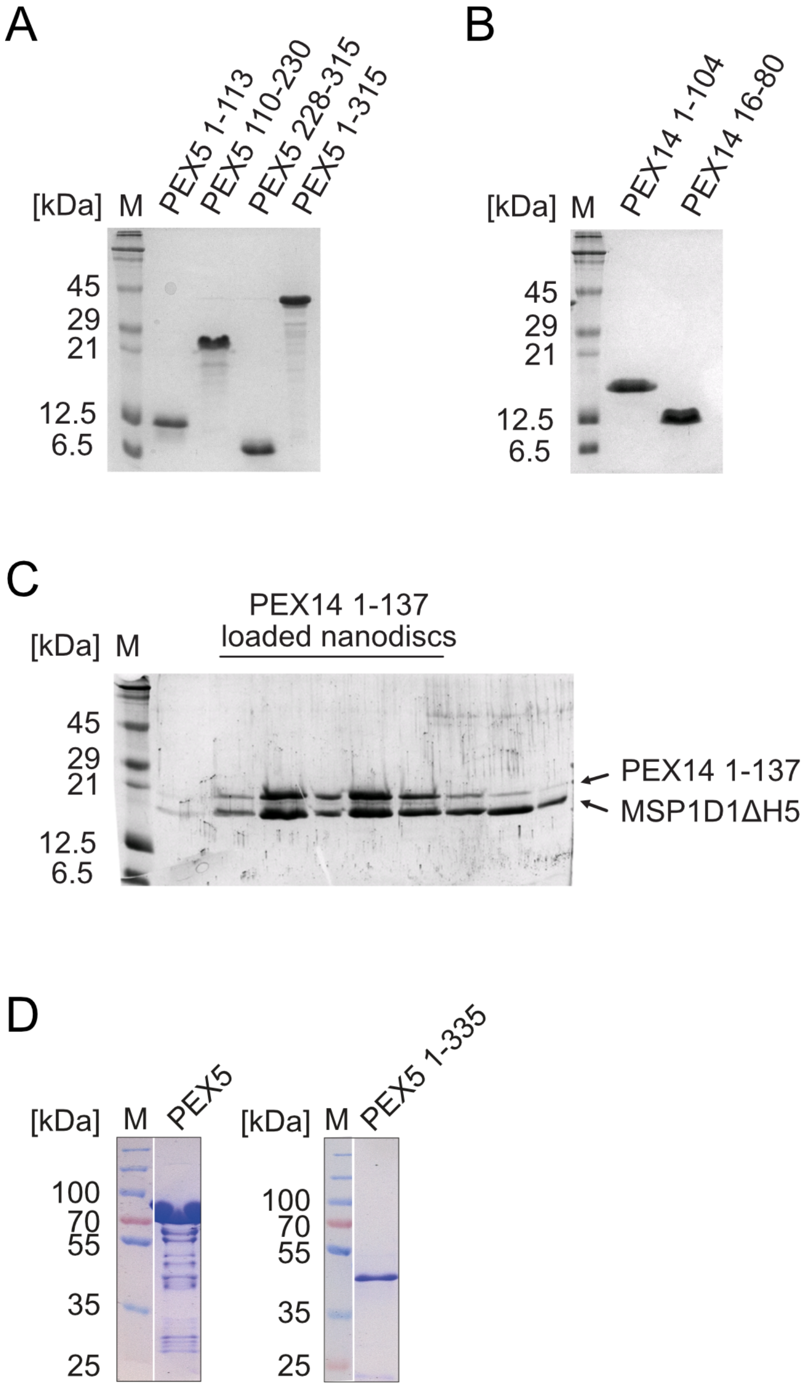

Supplementary Figure 5. SDS PAGE analysis of purified samples for (A) PEX5 1-113, 110-230, 228-315, 1-315 and (B) PEX14 1-104, 16-80. (C) SDS PAGE analysis of the size exclusion peak corresponding to PEX14 loaded nanodiscs. (D) SDS PAGE analysis of recombinant full-length PEX5 and PEX5 1-335 used for flotation assays. Note, that the degradation bands seen for PEX5 full-length do not bind to lipid vesicles in the flotation assay.
